# Hepatoprotective effects of mitoquinol mesylate in dogs with methylprednisolone acetate-induced steroid hepatopathy: A randomized crossover study

**DOI:** 10.14202/vetworld.2026.1356-1367

**Published:** 2026-03-28

**Authors:** Jevgenija Kondratjeva, Madara Nikolajenko, Aija Ilgaza

**Affiliations:** Preclinical Institute, Faculty of Veterinary Medicine, Latvia University of Life Sciences and Technologies, Jelgava, Latvia

**Keywords:** antioxidant therapy, dogs, glucocorticoids, hepatoprotection, mitoquinol mesylate, oxidative stress, steroid hepatopathy, veterinary medicine

## Abstract

**Background and Aim:**

Glucocorticoids are commonly used in veterinary medicine but often cause liver changes characterized by glycogen buildup, enzyme activation, and oxidative stress, known as steroid hepatopathy. Mitoquinol mesylate (MitoQ) is a mitochondria-targeted antioxidant that has shown hepatoprotective effects in experimental models; however, its potential benefits in dogs have not yet been studied. This research aimed to assess the hepatoprotective and antioxidant effects of MitoQ in dogs with experimentally induced methylprednisolone acetate (MPA)-related steroid hepatopathy.

**Materials and Methods:**

A randomized two-period crossover study was conducted using seven healthy adult Beagle dogs. Each treatment period lasted 28 days and was separated by a 28-day washout phase. In both periods, MPA (2 mg/kg, intramuscular) was administered on day 0 to induce steroid hepatopathy. Dogs received either MitoQ (20 mg/day/dog, orally) or a placebo once daily during each treatment period according to the crossover design. Clinical monitoring was performed daily. Blood samples were collected on days 0, 7, 14, 21, and 28 to measure alanine aminotransferase (ALT), alkaline phosphatase (ALP), corticosteroid-induced alkaline phosphatase (CIAP), and gamma-glutamyl transferase (GGT) activities. Liver biopsies were obtained on days 0, 14, and 28 for histopathological and immunohistochemical evaluation, including periodic acid–Schiff (PAS) staining and α-smooth muscle actin (α-SMA) detection. Data were analyzed using repeated-measures analysis of variance, and p < 0.05 was considered statistically significant.

**Results:**

MPA administration increased ALP, CIAP, and GGT activities in both groups, confirming steroid hepatopathy. Dogs receiving MitoQ exhibited lower enzyme elevations and a quicker return toward baseline compared to placebo-treated dogs, although not all differences reached statistical significance. Histological examination showed typical glycogen-type hepatocellular vacuolation in both groups, but lesions were generally milder in the MitoQ-treated group. PAS staining confirmed glycogen accumulation, and α-SMA immunostaining indicated only mild stellate cell activation, which tended to be lower during MitoQ treatment. No clinically relevant adverse effects were observed.

**Conclusion:**

MitoQ modestly reduced biochemical and histological changes linked to MPA-induced steroid hepatopathy in dogs and may serve as a promising adjunctive hepatoprotective therapy during glucocorticoid use. Larger controlled studies with longer follow-up periods and oxidative stress biomarkers are necessary to verify these initial results.

## INTRODUCTION

Glucocorticoids (GCs) are among the most frequently used medications in veterinary medicine. They are routinely prescribed to treat inflammatory, allergic, and autoimmune conditions, including atopic dermatitis, autoimmune hemolytic anemia, and inflammatory bowel disease in dogs and cats [1–3]. While GCs offer vital therapeutic benefits when used properly, their use is often linked to several adverse effects, especially with long-term or repeated treatment. The liver, being the primary organ responsible for steroid metabolism, is also a main target of GC-related changes [4, 5].

A well-known hepatic response to GC exposure in dogs is steroid (vacuolar) hepatopathy (SH). This condition features hepatocyte swelling, cytoplasmic vacuolization, nuclear marginalization, and excessive glycogen buildup within hepatocytes. The underlying mechanism involves increased glycogen synthase activity, heightened gluconeogenesis, and excessive glycogen storage. Additionally, GCs induce hepatic cytochrome P450 enzymes and stimulate the production of the corticosteroid-induced alkaline phosphatase (CIAP) isoenzyme [6–9].

Although these changes are generally reversible, ongoing hepatocellular stress can cause wider metabolic and oxidative problems. A retrospective study by Sepesy *et al*. [6] found that vacuolar hepatopathy often occurs alongside endocrine and inflammatory disorders. Persistent enzyme induction and oxidative damage may lead to liver dysfunction in dogs with iatrogenic hypercortisolism or repeated GC treatments.

Methylprednisolone acetate (MPA) is a long-acting injectable glucocorticoid ester commonly used because of its extended anti-inflammatory effects. A single injection can suppress inflammation for several weeks, making it helpful for patients with chronic allergic or pruritic conditions where owner compliance is limited. However, repeated use may lead to chronic hypercortisolism, causing sustained hepatic enzyme induction, glycogen buildup, and oxidative imbalances. Since the liver handles detoxification and metabolism of GCs, prolonged exposure can result in mitochondrial stress, lipid peroxidation, and depletion of the body’s natural antioxidant defenses [12, 13].

Oxidative stress (OS) is recognized as a key pathogenic mechanism in many canine liver diseases. Dogs suffering from chronic hepatitis, cholangitis, or endocrine hepatopathies display elevated levels of malondialdehyde and urinary F_2_-isoprostanes, signaling excessive free-radical production and inadequate antioxidant defenses. Mitochondria are both the main source and target of reactive oxygen species (ROS). When antioxidant capacity is overwhelmed, ROS cause lipid peroxidation, disrupt membrane potential, and lead to hepatocellular damage and apoptosis [10, 14, 15].

In clinical veterinary practice, hepatoprotective therapy is often used as supportive treatment in dogs with liver disease; however, strong evidence supporting many commonly used antioxidants or nutraceuticals, particularly for GC-associated hepatic alterations, remains limited [10, 11, 16].

Conventional hepatoprotectants, such as vitamin E and S-adenosyl-L-methionine (SAMe), offer only partial protection to mitochondria. As a result, recent focus has shifted toward mitochondria-targeted antioxidants (MTAs), which act directly at the site of oxidative damage [17, 18]. Mitoquinol mesylate (MitoQ) is one of the most well-studied MTAs. Structurally, it is a coenzyme Q_10_ analog attached to a lipophilic triphenylphosphonium cation, which allows it to accumulate within mitochondria several hundred times over through the mitochondrial membrane potential [17–19]. MitoQ neutralizes superoxide and peroxynitrite radicals, stabilizes mitochondrial membranes, and restores redox balance. Experimental studies have shown that MitoQ decreases lipid peroxidation, collagen deposition, and stellate cell activation in liver injury models, with safety confirmed through human clinical trials [19–25].

Despite the widespread use of GCs in canine clinical practice, effective strategies to prevent or reduce GC-associated hepatic alterations remain limited. Most current hepatoprotective approaches rely on conventional antioxidants or nutraceutical supplements that support overall antioxidant capacity but do not specifically target mitochondrial injury. Increasing evidence indicates that mitochondrial dysfunction plays a significant role in the development of SH, as prolonged GC exposure promotes glycogen buildup, metabolic imbalance, and excessive ROS production within hepatocytes. MTAs have demonstrated protective effects in experimental liver injury models in both rodents and humans; however, their potential use in dogs has not been thoroughly investigated. To our knowledge, no controlled experimental study has assessed the effect of MitoQ on GC-induced hepatic changes in dogs. Given the frequent use of GCs and the clinical importance of SH in canine patients, exploring mitochondria-targeted antioxidant therapy is warranted.

This study aimed to evaluate the hepatoprotective and antioxidant effects of MitoQ in dogs with MPA-induced SH using a randomized two-period crossover design. The study examined biochemical, histopathological, and immunohistochemical changes in the liver after GC administration and assessed whether treatment with MitoQ could lessen the severity of hepatic alterations compared to placebo. We hypothesized that administering MitoQ during GC exposure would reduce liver enzyme elevations, decrease hepatocellular vacuolation and glycogen buildup, and inhibit activation of hepatic stellate cells. Demonstrating such effects would support the use of MTAs as an additional hepatoprotective strategy in dogs receiving systemic GCs.

## MATERIALS AND METHODS

### Ethical approval

The experimental protocol was reviewed and approved by the Food and Veterinary Service of the Republic of Latvia under permit No. 70 for the use of animals in scientific procedures. All procedures were conducted in accordance with national legislation governing animal welfare and the protection of animals used for scientific purposes, as well as European Directive 2010/63/EU. The study was performed under institutional animal welfare and biosafety requirements.

Before inclusion in the experiment, all dogs underwent clinical examination and baseline hematological and serum biochemical evaluation to confirm their health status. Only clinically healthy animals were enrolled. Throughout the study, special attention was given to minimizing pain, stress, and discomfort. Dogs were acclimatized to housing and handling conditions before the start of the experiment and were maintained under controlled environmental conditions with daily monitoring, environmental enrichment, free access to water, and appropriate feeding.

All invasive procedures were performed by trained personnel using appropriate restraint, sedation, and local analgesia. Liver biopsy procedures were conducted under intravenous propofol sedation with local infiltration of lidocaine at the puncture site, and ultrasound guidance was used to reduce the risk of injury to major vessels, bile ducts, and the hepatic capsule. After biopsy, the animals were monitored clinically and ultrasonographically for early detection of complications. Humane endpoints were predefined, and any animal showing clinically relevant adverse effects or complications would have been withdrawn immediately from the study and provided with appropriate veterinary care. No animals met the withdrawal criteria during the study period. Every effort was made to ensure refinement of procedures, reduction in animal distress, and compliance with accepted ethical standards for animal experimentation.

### Study period and location

The study was conducted between April 10, 2015, and July 31, 2015 at the Preclinical Institute, Faculty of Veterinary Medicine, Latvia University of Life Sciences and Technologies, Jelgava, Latvia.

### Animals and their housing conditions

Seven 2-year-old, intact female Beagle dogs were housed at the Preclinical Institute, Faculty of Veterinary Medicine, Latvia University of Life Sciences and Technologies, Jelgava, Latvia. The dogs were kept individually at temperatures between 15°C and 21°C and relative humidity levels of 45% to 65%, under a natural light–dark cycle, and were fed a commercial lamb-and-rice diet twice daily. Their daily ration was adjusted according to the manufacturer’s recommendations based on body weight, with free access to water. Environmental enrichment included bedding, chew toys, daily human interaction, and visual and auditory contact with other dogs. The dogs had access to an enriched, secure outdoor enclosure during the daytime. The animals were acclimated to handling and experimental procedures before the start of the study.

All dogs were vaccinated and treated for endo- and ectoparasites following standard protocols. Before including them in the study, each animal underwent a thorough clinical examination to evaluate overall health. This assessment included checking body weight, body condition, skin and coat, superficial lymph nodes, mucous membranes, rectal temperature, and auscultation of the heart and lungs, along with baseline hematology and serum biochemistry tests. Only initially healthy animals with normal blood tests were selected for the study.

### Experimental treatments

A long-acting glucocorticoid, MPA (Depo-Medrol®, 40 mg/mL; Pfizer Inc., New York, NY, USA), was injected into the hindlimb muscles intramuscularly at a dose of 2 mg/kg. This dosage was chosen based on experimental studies and is known to provide sustained systemic glucocorticoid exposure sufficient to induce metabolic and hepatic responses without causing acute toxicity [1, 6, 12]. After intramuscular injection, peak pharmacodynamic effects are expected within the first week, with clinical effects lasting approximately 2–3 weeks, depending on individual variability [12, 13].

The antioxidant MitoQ (MitoQ®, Antipodean Pharmaceuticals Ltd., Auckland, New Zealand), supplied by the manufacturer in capsule form at a dose of 20 mg, was used as a hepatoprotective agent. The chosen fixed dose of 20 mg/day was based on previously published experimental studies demonstrating the biological activity and good tolerability of MitoQ *in vivo* [22–25], along with additional literature supporting the overall safety and antioxidant efficacy of mitochondria-targeted compounds [17–21].

Control capsules (placebo), identical in appearance and weight, contained only tapioca powder to verify the effect of the active compound. Capsules were stored at room temperature in their original packaging, protected from light and moisture, according to the manufacturer’s instructions. Personnel administering the treatments were not blinded; however, outcome assessors remained blinded throughout data collection and analysis.

### Experimental design

A crossover design with repeated-measures was employed in this study. Each dog acted as its own control, enhancing efficiency despite the small sample size. Because published data were insufficient to predict the expected effect of MitoQ in dogs, no formal *a priori* power calculation was conducted, and the study was designed as exploratory.

The experiment consisted of three phases: treatment (28 days), washout (28 days), and a second treatment (28 days). MPA (2 mg/kg, intramuscular) was given on day 0 of each of the two 28-day treatment periods. MitoQ or placebo was administered once daily for 28 days during each treatment phase, approximately at the same time each day.

The washout period of 28 days was chosen to be longer than the expected duration of clinical systemic activity after a single intramuscular MPA injection, which is typically around 3 weeks in dogs [26].

**Figure 1:**
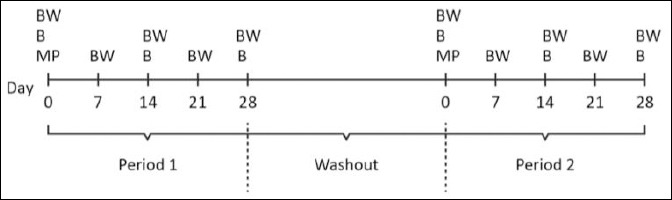
Study design and treatment sequence in a two-period crossover trial. MQ = mitoquinol mesylate treatment period, P = placebo treatment period.

Dogs were randomly assigned to two groups and allocated to two treatment sequences: group MQ received MitoQ, and group P received a placebo during the first period. After the washout, treatments were crossed over according to the crossover design (Figure 1).

The allocation sequence was created by someone not involved in outcome assessment using a simple random-number method. At the beginning of each treatment period, serum liver enzyme activities were within baseline values measured on day 0.

**Figure 2:**
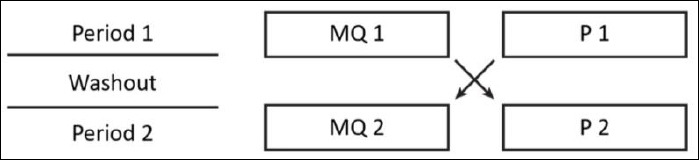
Sampling schedule and study timeline across the two-period crossover design. BW = bloodwork, B = liver biopsy, MP = methylprednisolone acetate injection.

Dogs were closely monitored daily during both treatment periods. Body weight was measured weekly. Adverse events were documented throughout the study. Blood samples were taken every 7 days (days 0, 7, 14, 21, and 28), and liver biopsies were performed every 14 days (days 0, 14, and 28) under ultrasound guidance during both treatment periods (Figure 2). Investigators conducting serum biochemical analyses, histological evaluations, and immunohistochemical assessments were blinded to treatment assignment.

### Blood sampling

For blood sample collection, the animals were manually restrained. Samples were drawn from the cephalic vein on the cranial side of the forelimb, just below the elbow joint. Before venipuncture, hair was clipped at the sampling site, and the skin was disinfected with 70% ethanol. Blood was collected using a 21G butterfly needle (Multifly® 21G; Sarstedt AG & Co. KG, Nümbrecht, Germany). A total of 6 mL of blood was drawn into serum tubes for biochemical analysis (Vacutest® Clot Activator; Vacutest Kima S. r.L., Arzergrande, Italy). After sampling, a pressure bandage was applied to the puncture site for 15 minutes. Samples were visually checked for hemolysis and lipemia before analysis; no clinically relevant interference was observed.

### Biochemical analysis of blood samples

Blood biochemical analyses were performed following standardized veterinary clinical pathology procedures. For serum separation, blood samples were centrifuged at 1300 rpm for 10 min, and serum analysis was conducted within 15 min after separation.

Serum activities of alanine aminotransferase (ALT), alkaline phosphatase (ALP), and gamma-glutamyl transferase (GGT) were measured using spectrophotometric detection on an automated clinical chemistry analyzer (Mindray BS-120; Mindray Bio-Medical Electronics Co., Shenzhen, China) with commercially available reagent kits (SPINREACT S. A., Girona, Spain). ALT, ALP, and GGT were assessed using kinetic photometric methods. Calibration and internal quality-control procedures were routinely carried out before sample analysis.

Assay precision was as follows: intra-assay CV% ranged from 0.36 to 1.11% for ALT, 0.35 to 0.41% for ALP, and 0.28 to 1.03% for GGT; inter-assay CV% ranged from 1.40 to 1.85% for ALT, 2.75 to 3.93% for ALP, and 1.16 to 2.05% for GGT. Routine measurements were performed with a single determination; samples were reanalyzed when results exceeded linearity limits or when quality-control performance necessitated repetition.

CIAP activity was measured after incubation at 65°C, followed by photometric assessment of residual CIAP activity [27].

### Liver biopsy procedure

Liver biopsies were performed following the recommendations of the World Small Animal Veterinary Association Liver Standardization Group [16]. Sedation was induced intravenously with propofol (Propofol®; Norbrook Laboratories Ltd., Peckforton, UK; 10 mg/mL) at 6 mg/kg. Subcutaneous infiltration of lidocaine (Lidocaine®; Grindeks JSC, Riga, Latvia; 20 mg/mL) at the planned puncture site, with an onset of 5–7 min, provided local anesthesia and analgesia.

The procedure was performed under ultrasound guidance to avoid damage to major vessels, bile ducts, and the hepatic capsule. To promote gallbladder emptying and lower the risk of perforation, 30 mL of olive oil was given orally to each dog 30 min before the biopsy. The dogs were positioned in right lateral recumbency, and the biopsy site was clipped and disinfected. Ultrasound examination was conducted using the MyLab30 Vet Gold system (Esaote S. p.A., Genoa, Italy) with a 5–7.5 MHz transducer.

A sterile, disposable 16-gauge Tru-Cut biopsy needle (Baxter International Inc., Deerfield, IL, USA) mounted on a biopsy gun (Vitesse® Biopsy Gun; OptiMed Medizinische Instrumente GmbH, Ettlingen, Germany) was used to obtain liver tissue. A small (~3 mm) skin incision was made before inserting the needle, which was advanced at least 1 cm into the liver parenchyma. Three passes were performed during each biopsy session.

Humane endpoints were established beforehand. After biopsy, dogs were monitored clinically and via ultrasound for 4 h to detect early complications (e.g., hemorrhage). Animals were to be withdrawn from the study and given appropriate veterinary care if any clinically significant adverse effects were observed. No animals met withdrawal criteria during the study.

### Preparation, fixation, and staining of liver tissue samples

During the experiment, 42 liver tissue samples were collected from seven dogs. All samples were processed using standard histological and immunohistochemical techniques. Liver specimens were immediately fixed in 10% neutral-buffered formalin (around 4% formaldehyde) for 24–48 h [16].

Dehydration and paraffin embedding were carried out using an automatic tissue processor (Citadel™ 69810040; Shandon Scientific Ltd., Cheshire, UK), following a standard protocol with graded alcohols (70%, 80%, 96%, and 100%) and xylene. Paraffin blocks were prepared using liquid paraffin (Histosec®; Merck KGaA, Darmstadt, Germany).

Sections of 4 μm and 3 μm thickness were cut using a microtome (SLEE Mainz Cut; SLEE Medical GmbH, Mainz, Germany) for histological and immunohistochemical analysis, respectively. Two sections from each sample were stained with hematoxylin and eosin (H&E) and periodic acid–Schiff (PAS). For PAS confirmation, adjacent sections underwent diastase digestion before PAS staining; the loss of PAS staining after digestion was interpreted as glycogen.

Additional sections were used for immunohistochemistry utilizing the Dako EnVision™ HRP system and a monoclonal mouse anti–α-smooth muscle actin (α-SMA) antibody (clone 1A4; Dako, Glostrup, Denmark) to identify activated hepatic stellate cells [8]. Heat-induced epitope retrieval was conducted in Target Retrieval Solution (pH 9; Dako) using a microwave oven (750 W) with three cycles of boiling followed by cooling.

The primary antibody was monoclonal mouse anti–α-SMA (clone 1A4; Dako), diluted 1:100 in Antibody Diluent (Dako), followed by EnVision+ HRP polymer anti-mouse (Dako). 3,3′-diaminobenzidine (DAB; Dako) was used as the chromogen, with hematoxylin for counterstaining. Vascular smooth muscle served as an internal positive control. Negative controls were prepared by omitting the primary antibody. Slides were examined under a light microscope (Leica DM5000B; Leica Microsystems GmbH, Wetzlar, Germany). Scoring was performed at ×200 magnification, and cellular counts were confirmed at ×400.

### Histological scoring

Hepatocyte morphological changes were semi-quantitatively graded into four categories across ten non-overlapping microscopic fields per sample (Table 1).

**Table 1:** Semi-quantitative scoring system for steroid hepatopathy in canine liver biopsies

Score	Category	Criteria	Image
0	No changes	No vacuolar degeneration was observed; hepatocyte cytoplasm appeared normal, and the nuclei remained centrally located.	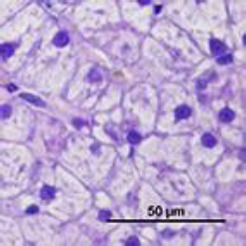
1	Mild changes	≤1/3 of hepatocytes affected within a microscopic field; reticular pattern with few or no vacuoles; predominantly central nuclei.	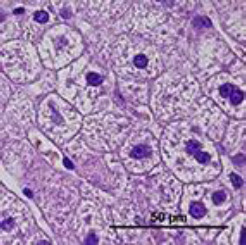
2	Moderate changes	≤2/3 of hepatocytes affected; reticular pattern with vacuolation; predominantly displaced nuclei toward the periphery.	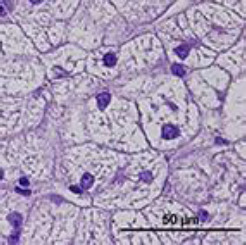
3	Severe changes	Nearly all hepatocytes affected; marked reticular pattern with variably sized vacuoles; predominantly peripheral nuclei.	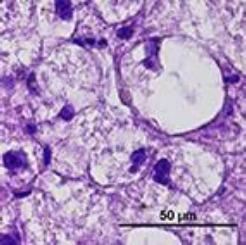

Activated hepatic stellate cells (α-SMA–positive) were quantified as the average cell count ± standard error per 100 hepatocytes [8]. Segmented neutrophil infiltration in hepatic parenchyma and vessels was evaluated in ten non-overlapping fields per sample as the average cell count ± standard error per microscopic field, and additionally categorized as follows:(1) none (negative), (2) 1–10 cells/field (low), (3) 10–20 cells/field (moderate), (4) 20–40 cells/field (high).

PAS-stained sections were evaluated for glycogen accumulation in ten microscopic fields per sample and graded as follows:(1) none (negative), (2) <1/3 of cells positive (mild), (3) up to 2/3 of cells positive (moderate), (4) nearly all cells positive (severe).

All histological assessments were conducted by a single investigator blinded to treatment allocation, and uncertain cases were re-evaluated to confirm scoring consistency.

### Statistical analysis

All statistical analyses were conducted using IBM SPSS Statistics version 28.0 (IBM Corp., Armonk, NY, USA). Data are presented as mean ± standard deviation (SD). The data were tested for normal distribution with the Shapiro–Wilk test and for equality of variances with Levene’s test. Differences between sampling times and treatment groups were assessed using repeated-measures analysis of variance, and when significant effects were found, Tukey’s post hoc test was used for pairwise comparisons.

Sphericity was evaluated using Mauchly’s test, and when the assumption was violated, the Greenhouse–Geisser correction was applied. Statistical significance was defined as p < 0.05. Since no data were missing, no imputation procedures were necessary.

## RESULTS

### Clinical monitoring and adverse effects

All seven dogs completed both crossover periods, and no missing data were recorded. No clinically relevant adverse events were observed during glucocorticoid exposure or MitoQ/placebo administration. Appetite and behavior remained unchanged in all animals, and no vomiting, diarrhea, polyuria, or polydipsia were noted. No injection-site reactions were observed.

### Biochemical findings

Administration of MPA caused increases in serum ALP, CIAP, and GGT activities, with a non-significant rise in ALT, which aligns with the biochemical profile of steroid hepatopathy. Unless specified otherwise, p values refer to comparisons with baseline (day 0) within each treatment period.

**Figure 3:**
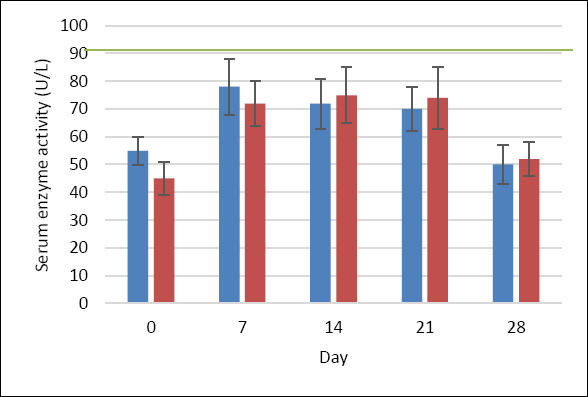
ALT activity (U/L) in dogs treated with MitoQ (group MQ, blue) or placebo (group P, red) during the treatment period. Data are presented as mean ± standard deviation. The upper reference limit for canine ALT (0–94 U/L) is indicated by the green horizontal line [28].

In the MQ group, serum ALT activity (reference range: 10–94 U/L [28]) showed a moderate increase after MPA injection, followed by a gradual decline toward baseline by day 28. A similar pattern was observed in the P group. No statistically significant differences were detected between groups or sampling days (Figure 3).

**Figure 4:**
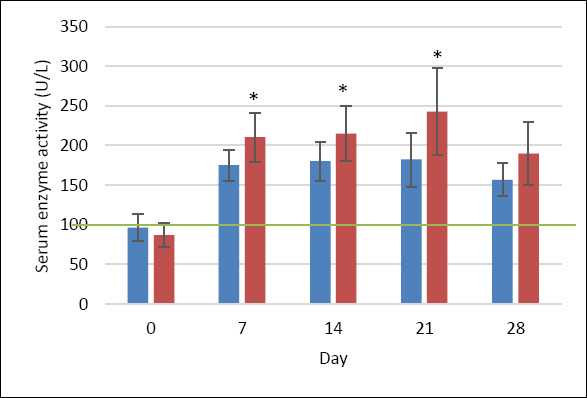
ALP activity (U/L) in dogs treated with MitoQ (group MQ, blue) or placebo (group P, red) during the treatment period. Data are presented as mean ± standard deviation. The upper reference limit for canine ALP (0–102 U/L) is indicated by the green horizontal line [28]. *p < 0.05 compared with baseline (day 0) within the same group.

ALP activity (reference range: 0–102 U/L [28]) in the MQ group increased 1.8-fold from baseline (96.5 ± 16.6 U/L) to day 7 (174.9 ± 19.6 U/L), remained relatively stable until day 21 (182.2 ± 34.1 U/L), and declined by day 28 (156.9 ± 20.7 U/L); these changes were not statistically significant. In contrast, the P group showed a statistically significant increase (p < 0.05), rising 2.4-fold by day 7 (210.2 ± 30.7 U/L), remaining elevated on day 14, and peaking at day 21 (242.9 ± 54.5 U/L) (Figure 4).

**Figure 5:**
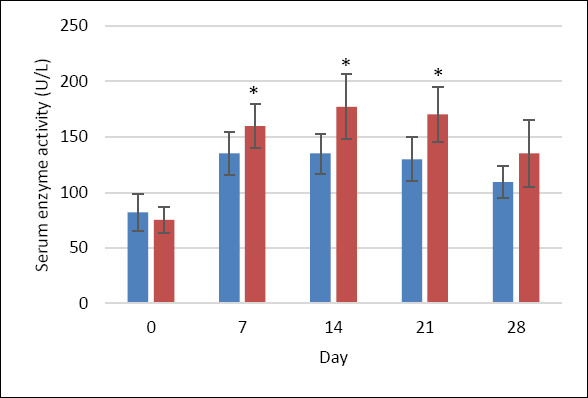
CIAP activity (U/L) in dogs treated with MitoQ (group MQ, blue) or placebo (group P, red) during the treatment period. Data are presented as mean ± SD. *p < 0.05 compared with baseline (day 0) within the same group.

CIAP activity showed a comparable trend. In the MQ group, CIAP increased 1.7-fold from baseline (81.8 ± 16.8 U/L) to day 7 (135.0 ± 19.0 U/L), stayed mildly elevated through day 21, and decreased to 109.1 ± 14.3 U/L by day 28. Differences were not statistically significant (p > 0.05). However, the P group exhibited significant increases (p < 0.05) at all time points, with a peak at day 14 (177.3 ± 29.1 U/L). Although neither group returned fully to baseline levels, dogs treated with MitoQ consistently showed lower enzyme activities (Figure 5). CIAP assessment was based on the heat-stable fraction of ALP (65 °C incubation) and was considered clinically meaningful only when combined with total ALP.

**Figure 6:**
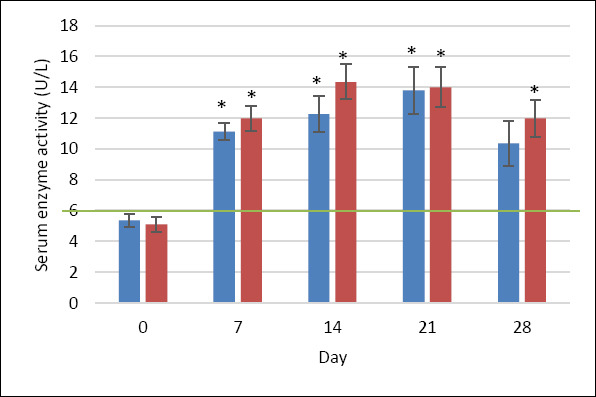
GGT activity (U/L) in dogs treated with MitoQ (group MQ, blue) or placebo (group P, red) during the treatment period. Data are presented as mean ± standard deviation. The upper reference limit for canine GGT (1–6 U/L) is indicated by the green horizontal line [28]. *p < 0.05 compared with baseline (day 0) within the same group.

GGT activity (reference range: 1–6 IU/L [28]) increased significantly in both groups after MPA administration. In the MQ group, GGT rose from 5.36 ± 0.44 U/L at baseline to 11.14 ± 0.54 U/L on day 7 (p < 0.05) and 12.26 ± 1.18 U/L on day 14 (p < 0.05), then declined to 10.37 ± 1.46 U/L by day 28 (p > 0.05). The P group showed more sustained increases (p < 0.05), peaking at day 14 with 14.36 ± 1.14 U/L. Although GGT levels remained slightly above normal limits at the end of the study, normalization happened earlier in the MQ group (Figure 6).

Overall, although some changes reached statistical significance, the observed pattern was consistent with steroid-induced enzyme induction (ALP/CIAP/GGT) rather than clinically relevant hepatocellular injury, as ALT changes were not statistically significant and remained within the reference interval. No clinically relevant adverse effects were observed.

### Histopathology

Histological examination of liver biopsy samples showed changes consistent with steroid hepatopathy in all dogs after MPA treatment. The main change was widespread hepatocellular vacuolation, often with nuclei displaced to the periphery and a finely reticulated cytoplasmic pattern. These features matched glycogen-type vacuolation, confirmed by PAS positivity and decreased staining after diastase digestion.

**Figure 7:**
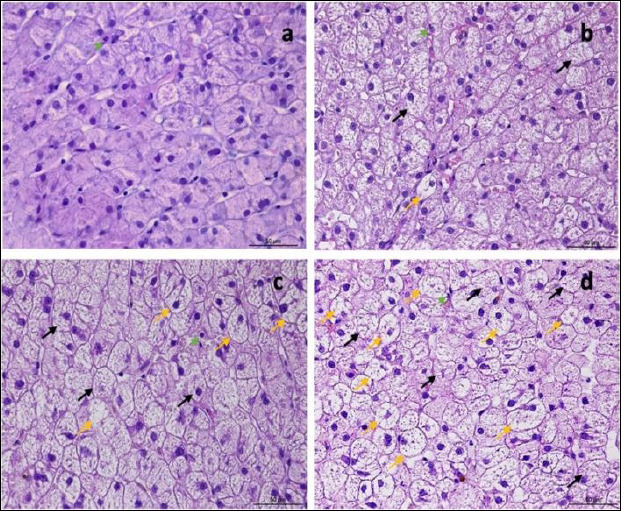
Representative images of normal liver histology (a) and steroid hepatopathy: (b) mild, (c) moderate, and (d) severe changes. A fine reticular cytoplasmic pattern (black arrows) with cytoplasmic vacuolation (yellow arrows) is observed. Neutrophils are present within the sinusoids (green arrow). H&E; original magnification ×400.

The P group exhibited more significant changes: by day 14, moderate to severe vacuolar degeneration of hepatocytes was observed and persisted through day 28. In several dogs in the P group, lesions remained severe throughout the study. In contrast, the MQ group showed milder hepatocellular alterations. On day 14, vacuolar degeneration ranged from mild to moderate, and by day 28, most samples exhibited partial recovery of hepatocyte architecture (Figure 7).

The inflammatory response was minimal in both groups. Neutrophil numbers increased slightly on day 14 (1–3 cells/field) and peaked on day 28 (up to 4 cells/field) without evidence of necrosis. No significant difference in inflammatory cell counts was observed between the groups.

The level of PAS positivity varied among animals in both MQ and P groups but generally matched the extent of hepatocellular vacuolation seen in hematoxylin–eosin sections. Vacuolar changes were consistent with glycogen storage and mild hydropic degeneration.

Overall, histological evaluation showed a typical steroid hepatopathy pattern in both groups, with less severe lesions in the MQ group, although the differences were not statistically significant.

### Immunohistochemistry

Mild activation of hepatic stellate cells was observed after MPA administration in both groups. In the MQ group, the number of α-SMA–positive stellate cells increased slightly from baseline (1–2 cells per 100 hepatocytes) to day 14 (4–6 cells) and remained similar on day 28.

In the P group, stellate cell counts rose from baseline levels (2–3 cells per 100 hepatocytes) to day 14 (5–8 cells), with little change by day 28 (4–6 cells). The overall increase remained mild in both groups, and there were no statistically significant differences between MQ and P groups (p > 0.05).

## DISCUSSION

### Effects of MPA on hepatic changes

A single injection of MPA caused the typical pattern of steroid hepatopathy in dogs, with the expected increases in ALP, CIAP, and GGT activities and the characteristic hepatocellular vacuolation indicating glycogen buildup. These results align with previous reports describing glucocorticoid-induced liver responses [6, 8, 12]. Although the biochemical changes were temporary, they suggest a phase of metabolic and OS within liver cells.

### Effects of MitoQ on biochemical and histological alterations

When MitoQ was administered, the response varied. Enzyme peaks were lower, and recovery seemed faster, especially for GGT and ALP. This effect might indicate protection of mitochondria from ROS, enabling hepatocytes to preserve membrane stability and metabolic functions [17–20]. Similar hepatoprotective effects of MitoQ have been reported in rodent models of toxin-induced liver injury [23–25], and the current findings suggest a comparable trend in dogs.

Microscopically, a similar qualitative trend was observed. Vacuolar degeneration was still present but less pronounced in the MQ group, and hepatocyte architecture appeared closer to normal by the end of the study. The inflammatory reaction remained minimal, supporting that the primary process was metabolic rather than inflammatory. Notably, α-SMA staining demonstrated only mild activation of stellate cells overall, but dogs receiving MitoQ showed slightly lower expression. This observation may indicate that mitochondrial protection reduces early fibrogenic signaling, as suggested in experimental fibrosis models [23, 25]. However, this inter-pretation should be made cautiously because of the small sample size and the semi-quantitative nature of the assessment.

### Comparison with conventional hepatoprotective therapies

In clinical veterinary practice, antioxidants and membrane-stabilizing agents such as S-adenosyl-L-methionine, silybin/silymarin, and vitamin E are frequently used as hepatoprotective agents in dogs. These compounds are recommended mainly as adjunctive options because they support antioxidant defense and hepatic glutathione homeostasis [29]. Experimental studies have demonstrated that nutraceutical formulations containing silymarin and vitamin E can enhance oxidative status and provide hepatoprotective effects [30]. However, controlled clinical evidence remains limited, and S-adenosyl-L-methionine continues to be the most commonly used cytoprotective and antioxidant option in canine hepatobiliary disease [29–31].

### Role of mitochondria-targeted antioxidant therapy

MitoQ is designed to accumulate within mitochondria and neutralize mitochondrial ROS more effectively than conventional antioxidants [17–21]. Because glucocorticoid exposure is linked to hepatic metabolic disturbances and OS, a mitochondria-targeted antioxidant approach may be especially relevant for steroid-associated liver changes [15, 17–21]. MitoQ could therefore be considered as an additional supplement in dogs needing prolonged systemic glucocorticoid therapy, such as for immune-mediated skin disorders or severe atopic dermatitis, where steroid-related side effects are a clinical concern [1, 4]. MitoQ has shown hepatoprotective effects in experimental models of liver injury, including inflammatory and fibrotic conditions [23–25], generally attributed to reducing mitochondrial OS and maintaining mitochondrial function [17, 19–21, 23, 24]. However, there are no direct comparative studies between MitoQ and other common hepatoprotective agents, and any potential benefits are largely based on mechanistic understanding.

### Safety considerations and clinical implications

MitoQ has been assessed in both rodent and human studies and is generally well tolerated, with no consistent evidence of hepatotoxicity or systemic adverse effects at doses comparable to or higher than those used in the present study [17–22]. However, controlled long-term data in dogs are limited, and future research should examine longer treatment durations in canine patients receiving chronic glucocorticoids, along with monitoring of liver enzymes and broader safety endpoints [29, 32].

Taken together, these findings support a connection between mitochondrial oxidative injury and the development of steroid hepatopathy. To our knowledge, no previous canine hepatoprotective study has combined a long-acting glucocorticoid model with serial biochemical evaluations, PAS-based glycogen confirmation, and α-SMA assessment within a two-period crossover design. The crossover approach enhanced the study’s internal validity and minimized inter-individual variability. Although limited in scope, the current results offer preliminary evidence supporting the use of MitoQ as a hepatoprotective supplement during or after glucocorticoid treatment in dogs. Larger, longer-term studies with different steroid and MitoQ dosing protocols are necessary for more definitive conclusions. Nonetheless, the present findings are promising and warrant further investigation.

### Limitations

The sample size was small, which might have decreased the statistical power to detect subtle differences between treatments. Although a two-period crossover design with a washout phase was employed to reduce inter-individual variability, period and carryover effects inherent in crossover studies cannot be entirely eliminated. A formal analysis of the time × treatment interaction was not conducted, and treatment effects were interpreted across different time points.

Follow-up was restricted to the 28-day observation window within each treatment period; therefore, long-term outcomes and the persistence of biochemical and histological changes could not be assessed. OS was evaluated indirectly through liver enzyme activities and histological findings, and no specific OS biomarkers, such as malondialdehyde or isoprostanes, were measured. Pharmacokinetic data for MitoQ and MPA were not collected, and exposure–response relationships could not be established. Additionally, only a single MPA dosing regimen was tested, which limits the ability to interpret dose–response relationships and apply findings to other corticosteroid protocols.

## CONCLUSION

Administration of MPA produced the expected biochemical and histological features of steroid hepatopathy in dogs, including increases in ALP, CIAP, and GGT activities, as well as hepatocellular vacuolation associated with glycogen accumulation confirmed by PAS staining. Treatment with MitoQ resulted in milder enzyme elevations, faster normalization of biochemical parameters, and less pronounced histological changes, along with slightly lower α-SMA expression, indicating reduced activation of hepatic stellate cells. These findings suggest that mitochondrial OS plays a role in the development of steroid hepatopathy and that mitochondria-targeted antioxidant therapy may help reduce these changes.

From a practical standpoint, the results support the potential use of MitoQ as an adjunctive hepatoprotective supplement in dogs receiving systemic glucocorticoids, especially in patients requiring repeated or long-acting corticosteroid administration, where hepatic enzyme induction and metabolic stress are common. The crossover design, repeated measurements, and combined biochemical, histological, PAS, and immunohistochemical assessments strengthen the reliability of these findings despite the small sample size. To the best of our knowledge, this is the first experimental veterinary study to evaluate the hepatoprotective effects of MitoQ in dogs with MPA-induced steroid hepatopathy.

However, the study was exploratory and limited to a short observation period, and OS was evaluated indirectly. Future research should involve larger sample sizes, longer treatment durations, direct measures of OS biomarkers, pharmacokinetic assessments, and comparisons with commonly used hepatoprotective agents to better understand the clinical benefits of mitochondria-targeted antioxidant therapy.

In conclusion, MitoQ demonstrated a moderate yet consistent hepatoprotective effect in dogs with MPA-induced steroid hepatopathy and may serve as a promising supportive strategy to lessen glucocorticoid-related hepatic changes. Additional controlled studies are necessary before recommending routine clinical use, but the current findings lay the groundwork for further research into MTA in veterinary hepatology.

## DATA AVAILABILITY

The supplementary data can be made available from the corresponding author upon request.

## AUTHORS’ CONTRIBUTIONS

JK and AI: Conceived and designed the study, drafted, edited, and revised the manuscript. JK and MN: Collected samples and data and performed data analysis and interpretation. MN: Conducted laboratory work and statistical analysis. AI: Supervised the study. All authors read and approved the final manuscript.

## References

[1] Viviano KR (2022). Glucocorticoids, cyclosporine, azathioprine, chlorambucil, and mycophenolate in dogs and cats: clinical uses, pharmacology, and side effects. Vet Clin North Am Small Anim Pract.

[2] Buriko Y, Tinsley A (2025). Controversies of and indications for use of glucocorticoids in the intensive care unit and the emergency room. Vet Clin North Am Small Anim Pract.

[3] Outerbridge CA, Jordan TJM (2021). Current knowledge on canine atopic dermatitis: pathogenesis and treatment. Adv Small Anim Care.

[4] Elkholly DA, Brodbelt DC, Church DB, Pelligand L, Mwacalimba K, Wright AK (2020). Side effects to systemic glucocorticoid therapy in dogs under primary veterinary care in the UK. Front Vet Sci.

[5] Tham HL, Davis JL (2024). Pharmacology of drugs used in autoimmune dermatopathies in cats and dogs: a narrative review. Vet Dermatol.

[6] Sepesy LM, Center SA, Randolph JF, Warner KL, Erb HN (2006). Vacuolar hepatopathy in dogs: 336 cases (1993–2005). J Am Vet Med Assoc.

[7] Oo T, Sasaki N, Ikenaka Y, Ichise T, Nagata N, Yokoyama N (2022). Serum steroid profiling of hepatocellular carcinoma associated with hyperadrenocorticism in dogs: a preliminary study. BMC Vet Res.

[8] Sobczak-Filipiak M, Szarek J, Czopowicz M, Mieczkowska J, Lechowski R (2014). Hepatic stellate cells in the liver of dogs with steroid-induced hepatopathy. Bull Vet Inst Pulawy.

[9] Tinted N, Pongcharoenwanit S, Ongvisespaibool T, Wachirodom V, Jumnansilp T, Buckland N (2023). Serum bile acids concentrations and liver enzyme activities after low-dose trilostane in dogs with hyperadrenocorticism. Animals (Basel).

[10] Bexfield N (2017). Canine idiopathic chronic hepatitis: diagnosis and treatment. Vet Clin North Am Small Anim Pract.

[11] Cullen JM (2009). Summary of the WSAVA liver standards. Vet Clin North Am Small Anim Pract.

[12] Tinklenberg RL, Murphy SD, Mochel JP, Seo YJ, Mahaffey AL, Yan Y (2020). Evaluation of dose-response effects of short-term oral prednisone administration on clinicopathologic and hemodynamic variables in healthy dogs. Am J Vet Res.

[13] Sebbag L, Mochel JP (2020). Pharmacokinetics of oral prednisone in dogs. Front Vet Sci.

[14] Phillips RK, Steiner JM, Suchodolski JS, Lidbury JA (2023). Urinary 15-F₂t-isoprostane concentrations in dogs with liver disease. Vet Sci.

[15] Perez-Montero B, Fermín-Rodriguez ML, Miró G, Cruz-Lopez F (2024). Oxidative stress in canine diseases: a comprehensive review. Antioxidants (Basel).

[16] Rothuizen J, Bunch SE, Charles JA, Cullen JM, Desmet VJ, Szatmári V (2006). WSAVA standards for clinical and histological diagnosis of canine and feline liver diseases.

[17] Jiang Q, Yin J, Chen J, Ma X, Wu M, Liu G (2020). Mitochondria-targeted antioxidants: a step towards disease treatment. Oxid Med Cell Longev.

[18] Murphy MP (2016). Understanding and preventing mitochondrial oxidative damage. Biochem Soc Trans.

[19] Oyewole AO, Birch-Machin MA (2015). Mitochondria-targeted antioxidants. FASEB J.

[20] Smith RAJ, Murphy MP (2011). Mitochondria-targeted antioxidants as therapies. Discov Med.

[21] Reily C, Mitchell T, Chacko BK, Benavides G, Murphy MP, Darley-Usmar V (2013). Mitochondrially targeted compounds and their impact on cellular bioenergetics. Redox Biol.

[22] Williamson J, Hughes CM, Cobley JN, Davison GW (2020). The mitochondria-targeted antioxidant MitoQ attenuates exercise-induced mitochondrial DNA damage. Redox Biol.

[23] Shan S, Liu Z, Liu Z, Zhang C, Song F (2022). MitoQ alleviates carbon tetrachloride-induced liver fibrosis in mice through regulating JNK/YAP pathway. Toxicol Res (Camb).

[24] Tao L, Xue YF, Sun FF, He X, Wang HQ, Tong CC (2024). MitoQ protects against carbon tetrachloride-induced hepatocyte ferroptosis and acute liver injury by suppressing mtROS-mediated ACSL4 upregulation. Toxicol Appl Pharmacol.

[25] Turkseven S, Bolognesi M, Brocca A, Pesce P, Angeli P, Di Pascoli M (2020). Mitochondria-targeted antioxidant mitoquinone attenuates liver inflammation and fibrosis in cirrhotic rats. Am J Physiol Gastrointest Liver Physiol.

[26] Depo-Medrone V 40 mg/ml Suspension for Injection Summary of Product Characteristics.

[27] Teske E, Rothuizen J, de Bruijne JJ, Rijnberk A (1989). Corticosteroid-induced alkaline phosphatase isoenzyme in the diagnosis of canine hypercorticism. Vet Rec.

[28] Willard MD, Tvedten H (2012). Small animal clinical diagnosis by laboratory methods.

[29] Webster CRL, Center SA, Cullen JM, Penninck DG, Richter KP, Twedt DC (2019). Diagnosis and treatment of chronic hepatitis in dogs: a consensus statement. J Vet Intern Med.

[30] Giannetto C, Arfuso F, Giudice E, Rizzo M, Piccione G, Mhalhel K (2022). Antioxidant and hepatoprotective effect of a nutritional supplement with silymarin phytosome, choline chloride, L-cystine, artichoke, and vitamin E in dogs. Antioxidants (Basel).

[31] Rodrigues A, Leal RO, Girod M, Dally C, Guery E, Gomes E (2020). Canine copper-associated hepatitis: a retrospective study of 17 clinical cases. Open Vet J.

[32] Habermaass V, Bartoli F, Gori E, Cogozzo A, Pierini A, Erba PA (2025). Serum liposoluble vitamins (A, D, E) in dogs with chronic biliary tract diseases versus healthy dogs. Vet Sci.

